# The *Drosophila eve* Insulator Homie Promotes *eve* Expression and Protects the Adjacent Gene from Repression by Polycomb Spreading

**DOI:** 10.1371/journal.pgen.1003883

**Published:** 2013-10-31

**Authors:** Miki Fujioka, Guizhi Sun, James B. Jaynes

**Affiliations:** Department of Biochemistry and Molecular Biology and the Kimmel Cancer Center, Thomas Jefferson University, Philadelphia, Pennsylvania, United States of America; New York University, United States of America

## Abstract

Insulators can block the action of enhancers on promoters and the spreading of repressive chromatin, as well as facilitating specific enhancer-promoter interactions. However, recent studies have called into question whether the activities ascribed to insulators in model transgene assays actually reflect their functions in the genome. The Drosophila *even skipped* (*eve*) gene is a Polycomb (Pc) domain with a Pc-group response element (PRE) at one end, flanked by an insulator, an arrangement also seen in other genes. Here, we show that this insulator has three major functions. It blocks the spreading of the *eve* Pc domain, preventing repression of the adjacent gene, *TER94*. It prevents activation of *TER94* by *eve* regulatory DNA. It also facilitates normal *eve* expression. When Homie is deleted in the context of a large transgene that mimics both *eve* and *TER94* regulation, *TER94* is repressed. This repression depends on the *eve* PRE. Ubiquitous *TER94* expression is “replaced” by expression in an *eve* pattern when Homie is deleted, and this effect is reversed when the PRE is also removed. Repression of *TER94* is attributable to spreading of the *eve* Pc domain into the *TER94* locus, accompanied by an increase in histone H3 trimethylation at lysine 27. Other PREs can functionally replace the *eve* PRE, and other insulators can block PRE-dependent repression in this context. The full activity of the *eve* promoter is also dependent on Homie, and other insulators can promote normal *eve* enhancer-promoter communication. Our data suggest that this is not due to preventing promoter competition, but is likely the result of the insulator organizing a chromosomal conformation favorable to normal enhancer-promoter interactions. Thus, insulator activities in a native context include enhancer blocking and enhancer-promoter facilitation, as well as preventing the spread of repressive chromatin.

## Author Summary

Insulators are specialized DNA elements that can separate the genome into functional units. Most of the current thinking about these elements comes from studies done with model transgenes. Studies of insulators within the specialized Hox gene complexes have suggested that model transgenes can reflect the normal functions of these elements in their native context. However, recent genome-wide studies have called this into question. This work analyzes the native function of an insulator that resides between the Drosophila genes *eve* and *TER94*, which are expressed in very different patterns. Also, the *eve* gene is a Polycomb (Pc) domain, a specialized type of chromatin that is found in many places throughout the genome. We show that this insulator has three major functions. It blocks the spreading of the *eve* Pc domain, preventing repression of *TER94*. It prevents activation of *TER94* by *eve* regulatory DNA. It also facilitates normal *eve* expression. Each of these activities are consistent with those seen with model transgenes, and other known insulators can provide these functions in this context. This work provides a novel and convincing example of the normal role of insulators in regulating the eukaryotic genome, as well as providing insights into their mechanisms of action.

## Introduction

A variety of regulatory elements have evolved in higher eukaryotes to regulate gene expression. Cis-regulatory modules (CRMs, or enhancers) are bound by DNA-binding transcription factors that coordinately recruit coactivators and corepressors. Enhancers communicate with basal promoters at least in part through a looping out of intervening DNA, allowing them to act over large distances along a chromosome, or even in *trans*, with a promoter on another chromosome [1]–[4]. Enhancer activities are regulated by the chromatin environment, which is “managed” by both the enhancers themselves and other DNA elements such as Polycomb-group response elements (PREs) [5]–[8]. Further coordination of these activities is provided by elements such as insulators that affect chromosomal organization and conformation. Insulators harbor activities that can limit the range of action of enhancers and repressive chromatin, as well as facilitate long-range enhancer-promoter communication, depending on context [9]–[12].

Insulators typically show “barrier” function that prevents the spread of heterochromatin, as well as enhancer blocking activity, in model transgene assays [9]–[12]. Pairs of insulators can interact with each other to generate chromosomal loops between them. This has been postulated to create distinct functional domains that somehow prevent enhancer-promoter cross-talk between domains.

Repressive chromatin structures include heterochromatin and Polycomb (Pc) chromatin, which constitutes a form of epigenetic transcriptional memory, stabilizing developmental fate choices, among other functions. Pc chromatin is maintained through the recruitment of Pc-group (PcG) gene products to PREs [5]–[8]. PREs can extend their influence outward to produce Polycomb domains that encompass multiple regulatory regions within a gene or a gene complex [13]–[17]. PREs can also synergize with each other in *trans* [18], and in some cases facilitate long-range enhancer-promoter communication [19]. Both Pc domains and mammalian X-inactivation involve the histone modification H3K27me3, catalyzed by Pc-repressive complex 2 (PRC2) [20]–[22].

The functions of PREs and insulators have been studied within Drosophila Hox genes [23]–[25]. There, functional chromatin domains are flanked by insulators, so that all the enhancers and PREs within a domain are coordinately regulated. Enhancers acting early in development (“initiators”) are spatially regulated to determine whether a domain will be active or not throughout the rest of development. They do this by inactivating PREs, so that where initiators are active, later-acting enhancers can also be active. The main effect of deleting insulators in this context is to extend the influence of initiators to inactivate PREs in the adjacent domain, which allows its later-acting enhancers to be inappropriately active. However, phenotypic details suggest that in some cells, repressive chromatin may spread instead [26].

Genome-wide chromatin immunoprecipitation (ChIP) analysis of the locations of insulator binding proteins show a wide range of binding patterns [27]–[40]. In *Drosophila*, insulator proteins include dCTCF (CCCTC-binding factor), Mod(mdg4)67.2, Su(Hw) (Suppressor of *hairy wing*), CP190 (centrosomal protein 190), BEAF32 (boundary element-associated factor 32), and Zw5 (Zeste-white-5). Recent genome-wide studies also implicate the mitotic spindle protein Chromator [41] and the nuclear lamina [30], [37] in insulator function. In mammals, CTCF is associated with most known insulators [10], [12], [42]–[44]. CTCF functions in the regulation of *β-globin* [45], [46] and the imprinted *Igf2* and *H19* loci [47]–[49]. Based on recent genome-wide studies, it has been suggested that insulator proteins bind at many sites that do not function as predicted by model transgene assays [34], [36], [38]. Transgenic dissection in a native context can help to determine their normal functions.

The *even skipped* (*eve*) locus is a well-defined Pc domain based on genome-wide analysis [13]–[17], and is regulated by PcG genes [50]–[54]. An insulator flanks its well-characterized regulatory region, which includes the *eve* PRE at its 3′ end [51], [55]. Thus, this insulator is in a position to separate both positive and negative *eve* regulatory elements from the constitutively expressed neighboring gene *TER94*, and/or to prevent ectopic activation of *eve* by *TER94* enhancers. This insulator was shown to have 3 distinct activities in model transgene assays. In addition to enhancer blocking, it causes homing of P-element transgenes to the endogenous *eve* neighborhood, for which it was nicknamed Homie (Homing insulator at *eve*). Furthermore, from within a several megabase region flanking endogenous *eve*, it causes long-range interactions of transgenic promoters with endogenous *eve* enhancers [55]. Genome-wide analysis showed that most known insulator proteins bind to the Homie region [27], [33].

Homie shares properties with other insulators based on model transgene assays and, like many other putative insulators, is situated close to both a transcription start site (TSS) and a PRE. Thus, understanding Homie's function in its native context can illuminate many of the mysteries that surround this enigmatic group of regulatory elements. In order to investigate its native function, we constructed a transgenic *eve-TER94* locus that mimics the normal regulation of both genes. Using this artificial locus, we show that Homie functions as a PRE blocker to protect *TER94* from repression due to spreading of the *eve* Pc domain. Heterologous insulators and PREs can substitute for Homie and the *eve* PRE, suggesting that limiting the range of PRE action is an important function of insulators generally. Homie also prevents the *eve* enhancers from activating *TER94* in specific tissues. Furthermore, Homie facilitates normal *eve* expression by augmenting communication between the *eve* promoter and its 3′ enhancers, likely through a chromosomal looping mechanism.

## Results

Insulators are generally considered to have two major functions. First, they can shield promoters from the effects of distal enhancers. Second, they can block the spread of repressive chromatin. Here, we investigate the roles that these activities play in the normal functions of an insulator (Homie) located between the ubiquitously expressed *TER94* gene and the highly patterned *eve* gene. We find that blocking the spread of repressive Polycomb chromatin by Homie is critical for normal *TER94* promoter expression. In addition to exhibiting the canonical insulator activities in a near-native context, we find that Homie facilitates certain aspects of normal *eve* expression, and we present a model for how this occurs.

### Homie Shields the *TER94* Promoter from *eve* PRE Activity

**Figure 1:**
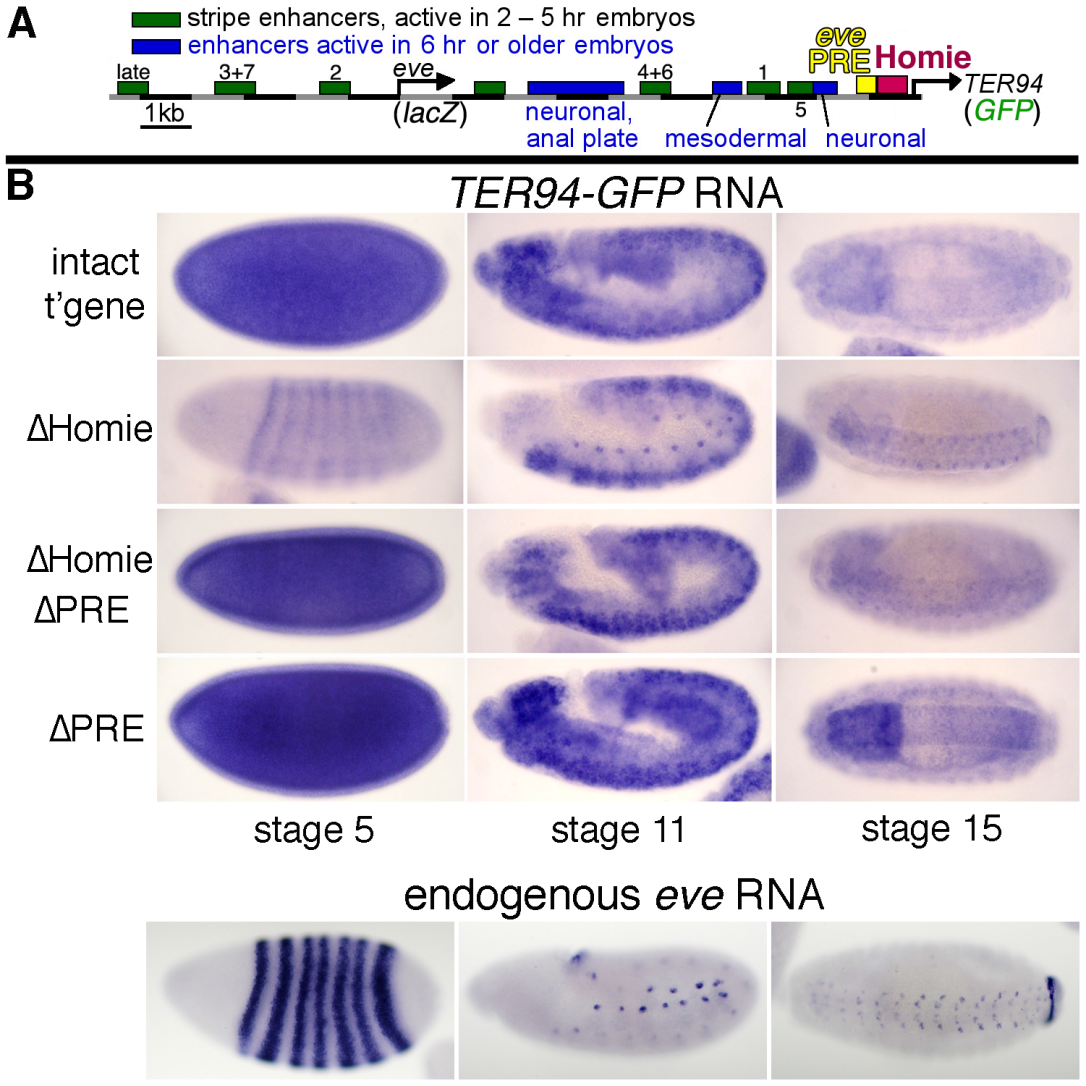
Homie shields the *TER94* promoter from *eve* PRE activity. **A:** map of the *eve-TER94* transgene. The 3^rd^ exon of the *TER94* protein coding region is fused to that of GFP, while the *eve* coding region is replaced by that of *lacZ*. Otherwise, the transgene consists of the entire genomic region from −6.4 kb to +11.3 kb relative to the *eve* TSS, and includes all of the *eve* enhancers, plus *TER94* enhancers located within its first two introns. The locations of the major *eve* PRE (“*eve* PRE”), 3′ insulator (“Homie”), early embryonic stripe enhancers (numbered), and late embryonic enhancers (labeled) are shown as colored boxes. **B:** embryonic expression, at the indicated stages, of GFP RNA, driven by the *TER94* promoter, from the transgene shown in A (top row “intact t'gene”), or the same transgene modified by deletion of either the insulator alone (2^nd^ row “ΔHomie”), the PRE alone (bottom row “ΔPRE”), or both (3^rd^ row “ΔHomie ΔPRE”), each inserted at attP landing site 95E5, visualized by whole-mount *in situ* hybridization. Note that the normal, ubiquitous expression (mimicking *TER94*) is changed to resemble the *eve* pattern (shown for comparison at the bottom) by deletion of Homie, while further deletion of the PRE restores *TER94*-like expression. Deletion of the PRE alone does not noticeably affect the embryonic pattern.

In order to analyze the function of the *eve* 3′ insulator Homie, we employed a pseudo-locus that contains all the regulatory DNA necessary for normal expression of both *eve* [56]–[59] and the 3′ adjacent gene *TER94* [60]–[62]. This transgene extends from −6.4 to +11.3 kb relative to the *eve* TSS, from the 5′-most enhancer of *eve* to the 3^rd^ exon of *TER94*. In addition to all of the *eve* enhancers, this region contains a characterized PRE [51] located just upstream (on the *eve* side) of Homie [55]. On the other side of Homie is the *TER94* promoter and TSS, which are sufficient for ubiquitous expression, augmented by enhancers in the *TER94* introns (data not shown). The *eve* coding region was replaced with *lacZ* coding DNA, and the 3^rd^ exon of *TER94* was fused with the EGFP coding region (Figure 1A). In this study, we make repeated use of a version of recombinase-mediated cassette exchange (RMCE) [63] that allows modified transgenes to be inserted in either orientation at pre-defined chromosomal landing sites. All aspects of transgene expression were consistent for both orientations and at multiple landing sites, with a few minor exceptions (as noted below).

In embryos, *TER94* RNA is present ubiquitously at early blastoderm, and begins to fade around stage 10. Most of this RNA is maternally derived, but there is a ubiquitous zygotic contribution as well (see below). At stage 10 and later, strong expression is also observed throughout the brain and central nervous system (CNS) [55]. *TER94-GFP* expression from our transgene simulates endogenous *TER94* expression (Figure 1B, “intact t'gene”). Although the level of expression varies somewhat with chromosomal location, the relative behavior of modified transgenes was consistent at each chromosomal location (compare Figure 1B and Figure S1).

Deletion of Homie caused a severe loss of early, ubiquitous expression driven by the *TER94* promoter (Figure 1B “ΔHomie”, Figure S1). When the *eve* PRE was deleted in addition to Homie, the ubiquitous expression in embryos returned (Figure 1B “ΔHomie ΔPRE”, Figure S1). Deletion of the *eve* PRE alone did not affect the expression pattern (Figure 1B “ΔPRE”). These results show that the loss of ubiquitous expression from the *TER94* promoter caused by deletion of Homie depends on the presence of the PRE. So, one function of Homie is to protect *TER94* from PRE-dependent repression.

We also note that when Homie is removed, expression in an *eve*-like pattern is seen (Figure 1B “ΔHomie”, Figure S1A). This indicates that without Homie, *eve* enhancers can access the *TER94* promoter. We investigate this effect further below.

### Homie Blocks *eve* PRE Activity in Ovaries

**Figure 2:**
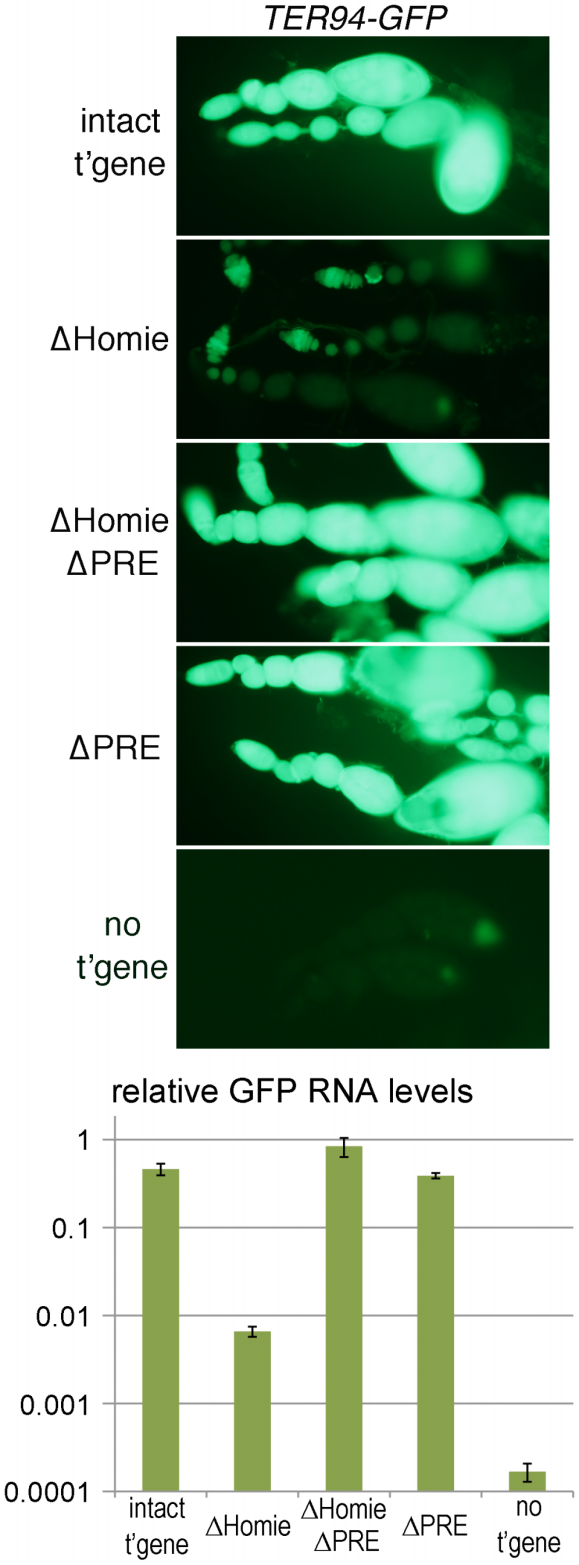
Homie blocks PRE action in ovaries. Fluorescence and GFP RNA levels in ovarioles from fly lines carrying the indicated transgenic reporters (described in Figure 1), or no transgene (“no t'gene”). Note the strong fluorescence from *TER94* promoter-driven GFP with the intact transgene (“intact t'gene”) at all stages of oogenesis (which proceeds from left to right within each string of ovarioles), and that this is lost when Homie is deleted (“ΔHomie”). Remarkably, strong GFP expression is restored when both Homie and the PRE are deleted (“ΔHomie ΔPRE”), indicating that in the absence of Homie, the PRE is responsible for repression of *TER94-GFP*. Strong expression is also seen when only the PRE is removed (“ΔPRE”). The graph at the bottom shows, on a log scale, the results of quantitation in triplicate (averages with standard deviations) of GFP RNA from ovaries of lines carrying the indicated transgenes (see Materials and Methods). Note that GFP RNA levels decrease more than 50-fold when Homie is deleted, and are restored by additional deletion of the PRE.

Early, ubiquitous expression of *TER94* comes from maternally deposited RNA, based on its early appearance and the fact that *TER94* is expressed strongly in developing oocytes [60]–[62]. This was confirmed by staining for transgene expression in the absence of a maternal contribution, which is much weaker at early stages than the maternally derived signal (Figure S2 “intact t'gene”; compare to Figure 1B, Figure S1). Since *TER94-GFP* RNA is deposited maternally, we examined expression in ovaries. *TER94* mRNA is present in both the germline, including nurse cells, and somatic epithelial follicle cells [60]–[62]. No *eve* expression in ovaries has been reported. In our transgenic lines, strong *TER94-GFP* expression was seen at all stages of oogenesis (Figure 2 “intact t'gene”, Figure S4) in both germline and somatic epithelial cells (Figure S3 “intact t'gene”). However, the level depended to some extent on chromosomal location (compare Figure 2 and Figure S4). In each case, expression was severely repressed when Homie was deleted (Figure 2 “ΔHomie”, Figure S4). As was seen in embryos, it was restored when the PRE was also deleted (Figure 2 “ΔHomie ΔPRE”, Figure S4). These data confirm that in ovaries, Homie is required for *TER94* promoter activity, due to its blocking of PRE-dependent repression.

### The *eve* PRE Is Redundant in Embryos

Since most of the ubiquitous *TER94-GFP* RNA seen in early embryos is maternally derived, we tested whether zygotic expression from a paternally-derived transgene is affected by Homie deletion. In this assay, non-transgene-carrying (*yw*) female flies are crossed with transgene-carrying males, so that there is no maternal GFP RNA in the progeny. Two chromosomal locations were analyzed. In both cases, GFP expression was reduced when Homie was deleted (Figure S2).

Because of the relatively low level of expression, we quantified GFP RNA using RT-PCR. Embryos from three timed collections were analyzed: 2–3 hr. (stages 5–6) and 4–6 hr. (stages 9–11) after egg deposition, and stages 13–15. The effect of Homie deletion paralleled those described above for both ovaries and embryos, in that expression was repressed. However, unlike in ovaries, when both Homie and the PRE were deleted, *TER94-GFP* expression remained repressed at all stages examined (Figure S2 and data not shown). This is consistent with the idea, confirmed below, that another PRE in the *eve* locus substitutes in embryos (but not in ovaries) for the *eve* 3′ PRE. In fact, the *eve* promoter-proximal region has PRE-like properties [51] (see Discussion).

### Other Drosophila Insulators Block *eve* PRE Action

**Figure 3:**
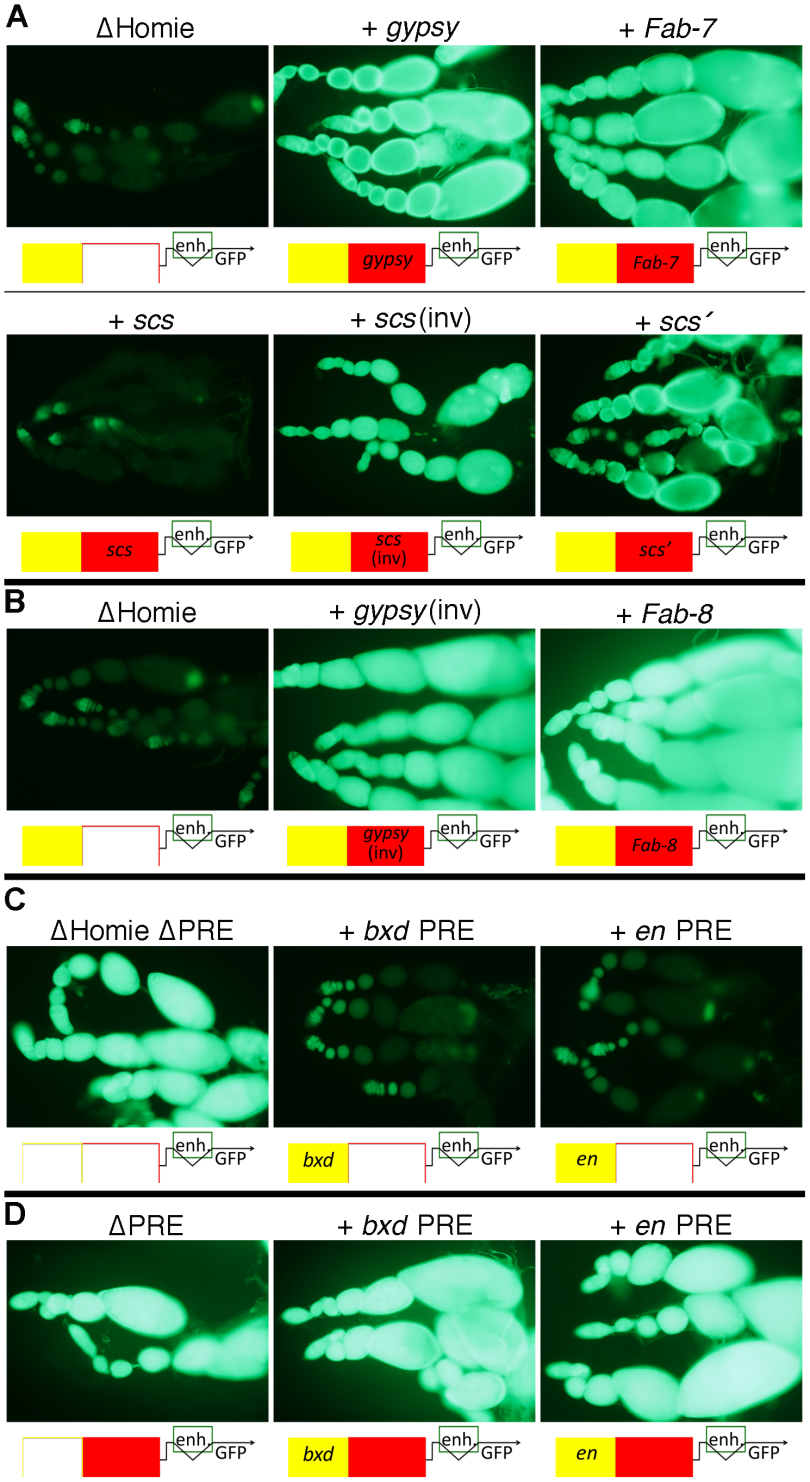
Other Drosophila insulators block *eve* PRE action, and other PREs are blocked by Homie. **A:** fluorescence from transgenic *TER94-GFP* in the context of the transgene diagrammed in Figure 1A at the 95E5 landing site, with Homie replaced by either λ phage DNA (“ΔHomie”) or the indicated insulator. Diagrams below each panel show the arrangement of regulatory elements affecting GFP expression: yellow-filled boxes are PREs, either from *eve* (unlabeled), *en*, or the *bxd* region of *Ubx*, as indicated; red-filled boxes are insulators, either Homie (unlabeled), or as labeled. Note that each of these insulators restore *TER94* promoter activity, although *scs* does so when inserted in one orientation (“*scs*(inv)”) but not the other (“*scs*”) relative to the *TER94-GFP* promoter. Note also that restoration of GFP expression is somewhat weaker for *scs'* and *scs* (even in the “inverted” orientation) than for *gypsy* and *Fab-7*, indicating that they only partially block *eve* PRE action. **B:** same as in A, except that the transgenes were inserted in opposite orientation at the same landing site relative to that in A, with either the *gypsy* insulator or the *Fab-8* insulator. Note that *eve* PRE action is blocked in both cases, causing strong fluorescence. **C:** fluorescence from transgenic *TER94-GFP* in the context of the transgene diagrammed in Figure 1A, with Homie deleted, and with either the *eve* PRE deleted (“ΔHomie ΔPRE”), or replaced by the *bxd* PRE (“+ *bxd* PRE”) or the *en* PRE (“+ *en* PRE”). Note that each of these PREs repress *TER94-GFP* to a similar degree as does the *eve* PRE in the absence of an insulator (compare to “ΔHomie” panels in A and B, where the *eve* PRE is present). **D:** fluorescence from transgenic *TER94-GFP* in the context of the transgene diagrammed in Figure 1A, with the *eve* PRE either removed (“ΔPRE”), or replaced by either the *bxd* PRE (“+ *bxd* PRE”) or the *en* PRE (“+ *en* PRE”). Note that neither of these PREs is able to repress *TER94-GFP* when Homie is present, showing that Homie blocks the repressive action of these heterologous PREs.

Are the functions of Homie seen in our assays unique, or are they shared among insulators? In order to test this, we replaced Homie with other known insulators. As a negative control, a >500 bp stretch of λ phage DNA was tested. It had no effect on repression of the *TER94* promoter by the *eve* PRE (Figure 3A “ΔHomie”). In contrast, other characterized Drosophila insulators can substitute for Homie to block repression. *gypsy* (Figure 3A, B), *Fab-7*, *scs'* (Figure 3A), and *Fab-8* (Figure 3B) each prevented *TER94* promoter repression. Although in one orientation, *scs* did not work (Figure 3A, “+ *scs*”), it did work in the opposite orientation (Figure 3A, “+ *scs*(inv)”). *Fab-8* and *gypsy* showed a minor directionality in their effectiveness (not shown). Restoration of GFP expression is somewhat weaker for *scs'* and *scs*(inv) than for *gypsy* and *Fab-7*, indicating that they only partially block *eve* PRE action. Despite differences in efficiency, blocking of PRE action in this context is a shared property of insulators.

### Heterologous PREs Repress *TER94* in the Absence of an Insulator, and Homie Blocks this Repression

In order to test whether the repression of *TER94* by the *eve* PRE is due to some unusual property associated with this PRE, we replaced it with other known PREs. We tested both the *bxd* PRE and an *en* PRE for the ability to substitute for the *eve* PRE in ovaries, in the context of a Homie-deleted transgene. In both cases, repression was seen at a comparable level to that seen with the *eve* PRE (Figure 3C, compare to Figure 3A,B “ΔHomie”), indicating that *TER94* repression is due to a property shared by PREs.

We also tested whether Homie can prevent repression by heterologous PREs. To so this, we replaced the *eve* PRE with either the *bxd* PRE or the *en* PRE. In both cases, Homie blocked their action on the *TER94* promoter, and the resulting GFP expression was like that of the wild-type transgene (Figure 3D). This shows that Homie can block repression by a variety of PREs. Taken together, these results suggest that insulators block PRE-dependent repression generally. Thus, the commonly occurring arrangement of PREs flanked on one side by insulators [31] is likely to function to provide a sharp transition in chromatin structure.

### Homie Prevents Spreading of the *eve* Polycomb Domain

**Figure 4:**
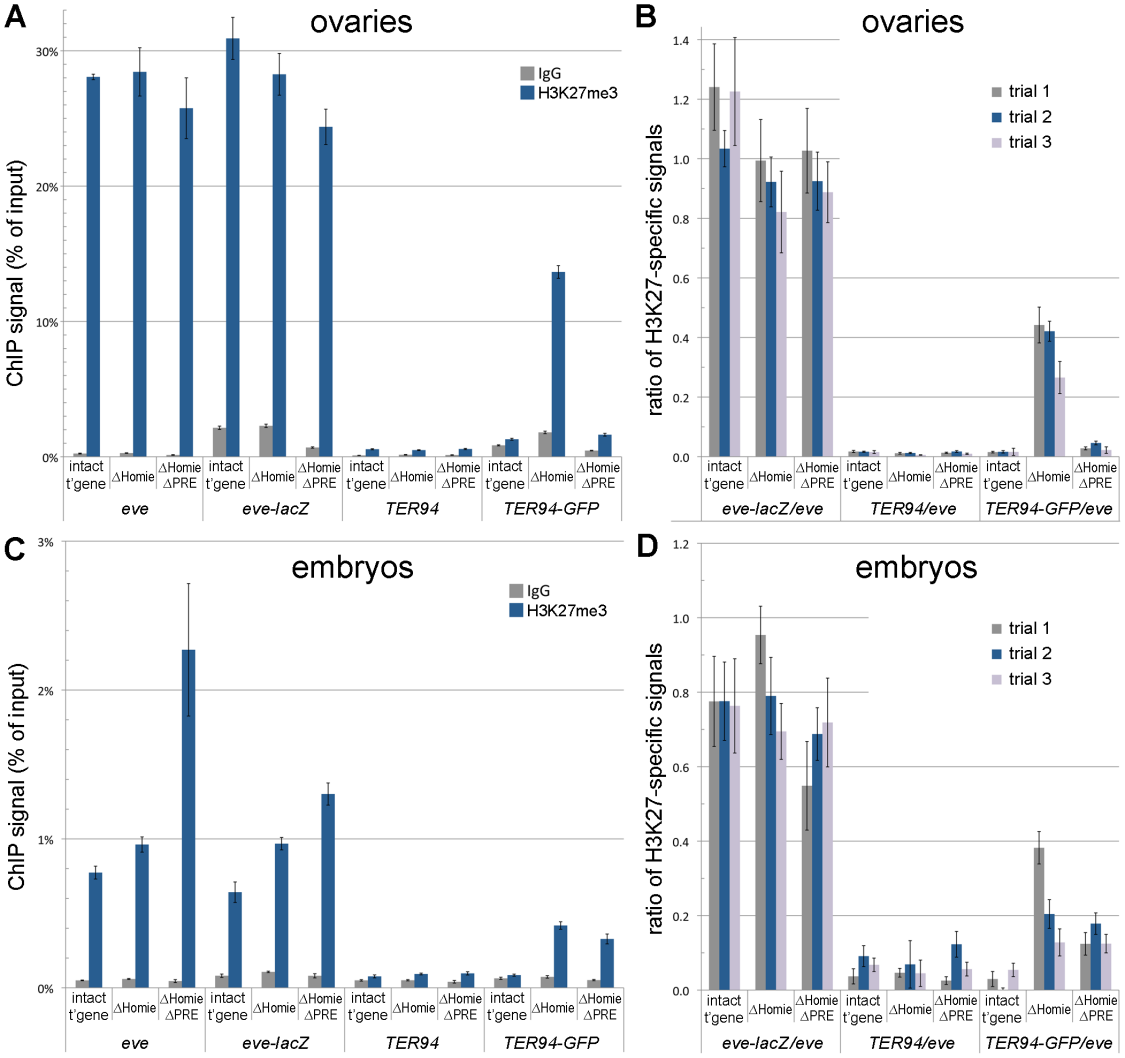
Homie blocks spreading of the *eve* Pc/H3K27me3 domain into the adjacent gene, *TER94*. ChIP assays were used to quantify the association of H3K27me3 with the endogenous *eve* and *TER94* coding regions, and with the transgenic *eve-lacZ* and *TER94-GFP* coding regions. **A, B:** ovaries were dissected and subjected to ChIP analysis, as described in Materials and Methods. In A is graphed the averages and standard deviations from triplicate PCR assays using primer pairs specific to each of the 4 coding region indicated along the bottom, and for the transgenic lines indicated (“intact t'gene” carries the entire transgene diagrammed in Figure 1A; “ΔHomie” is the same transgene with Homie deleted; “ΔHomie ΔPRE” has both Homie and the PRE deleted). Either H3K27me3-specific antibodies or non-specific IgG were used to precipitate cross-linked chromatin, as indicated in the inset key. In B is graphed the same data (“trial 2” in the inset key), and two other trials (“trial 1” and “trial 3”) using independent chromatin preparations (see Materials and Methods), normalized to the H3K27me3-specific signal from the *eve* coding region for each transgenic line. **C, D:** embryos (2–20 hr. after egg deposition) were collected and subjected to ChIP analysis, as in A and B above. Note that the specific signals from the transgenic *eve-lacZ* coding region do not change significantly when Homie is deleted, while the transgenic *TER94-GFP* coding region shows an increase in H3K27 trimethylation when Homie is deleted, correlating with its repression.

The *eve* locus is a Pc domain, associated with both Polycomb and the characteristic histone modification H3K27me3 [13]–[17]. We asked whether the repression of *TER94-GFP* in the ΔHomie transgene is accompanied by spreading of this Pc domain over the *TER94-GFP* promoter. Indeed, in ovaries, we found that H3K27me3 was increased in the *TER94-GFP* region when Homie was deleted (Figure 4A,B). Additionally removing the PRE reversed this effect almost completely (Figure 4A,B), indicating that spreading of H3K27me3 depends on the *eve* PRE. Thus, when *TER94-GFP* is repressed, H3K27me3 is increased, and when this repression is reversed, H3K27me3 levels return to normal. This suggests that Pc domain spreading is likely to be responsible for the repression.

In embryos, as in ovaries, H3K27me3 spreads into the *TER94-GFP* region when Homie is deleted (Figure 4C,D). However, in contrast to ovaries, additionally removing the PRE does not reverse the effect (Figure 4C,D), suggesting that there is redundancy between this PRE and other PREs in embryos. This redundant activity may be provided by the *eve* upstream promoter region [51], or by uncharacterized PREs within the *eve* locus. Again, recalling that the *eve* PRE is redundant in embryos for repression of *TER94-GFP* in the absence of Homie (Figure S2), there is a striking correlation between spreading of the Pc domain and repression of the *TER94* promoter.

### Homie Shields the *TER94* Promoter from *eve* Enhancers

Intriguingly, when Homie is deleted, the loss of ubiquitous *TER94-GFP* expression is accompanied by weak expression in an *eve* pattern (Figure 1B “ΔHomie”, Figure S1A). With the intact transgene, early stripe expression of *TER94-GFP* driven by *eve* enhancers might be obscured by early ubiquitous expression, so we cannot rule out that *eve* enhancers are working on the *TER94* promoter at early stages. In fact, *eve*-like stripe expression from the transgenic *TER94* promoter is seen at one chromosomal landing site when the intact transgene is heterozygous and paternally derived, so that there is no maternal contribution (Figure S2B “intact t'gene”). However, *eve*-like mesodermal, CNS, and anal plate ring (APR) expression seems clearly to be caused by deletion of Homie (Figure 1B “ΔHomie”, stages 11 and 15; Figure S1A “ΔHomie”, stage 13), because with the intact transgene, ubiquitous expression in these tissues is low, yet no such *eve*-like expression is seen. Furthermore, these later-stage aspects of *eve* expression are not seen with a paternally derived, intact transgene (Figure S2). Therefore, the data suggest that one of Homie's functions is to prevent interaction between the *TER94* promoter and *eve* enhancers. Accompanying the recovered ubiquitous expression when the PRE is also deleted, expression in an *eve* pattern is lost (Figure 1B “ΔHomie ΔPRE”, stages 11 and 15; Figure S1A “ΔHomie ΔPRE”, stage 13). This loss of mesodermal, CNS, and APR expression of *TER94-GFP* caused by additional deletion of the PRE indicates that the PRE not only represses ubiquitous *TER94* promoter activity, but also facilitates communication between the *eve* enhancers and the *TER94* promoter in the absence of Homie (see Discussion).

### Homie Facilitates *eve* 3′ Enhancer Action on the *eve* Promoter

**Figure 5:**
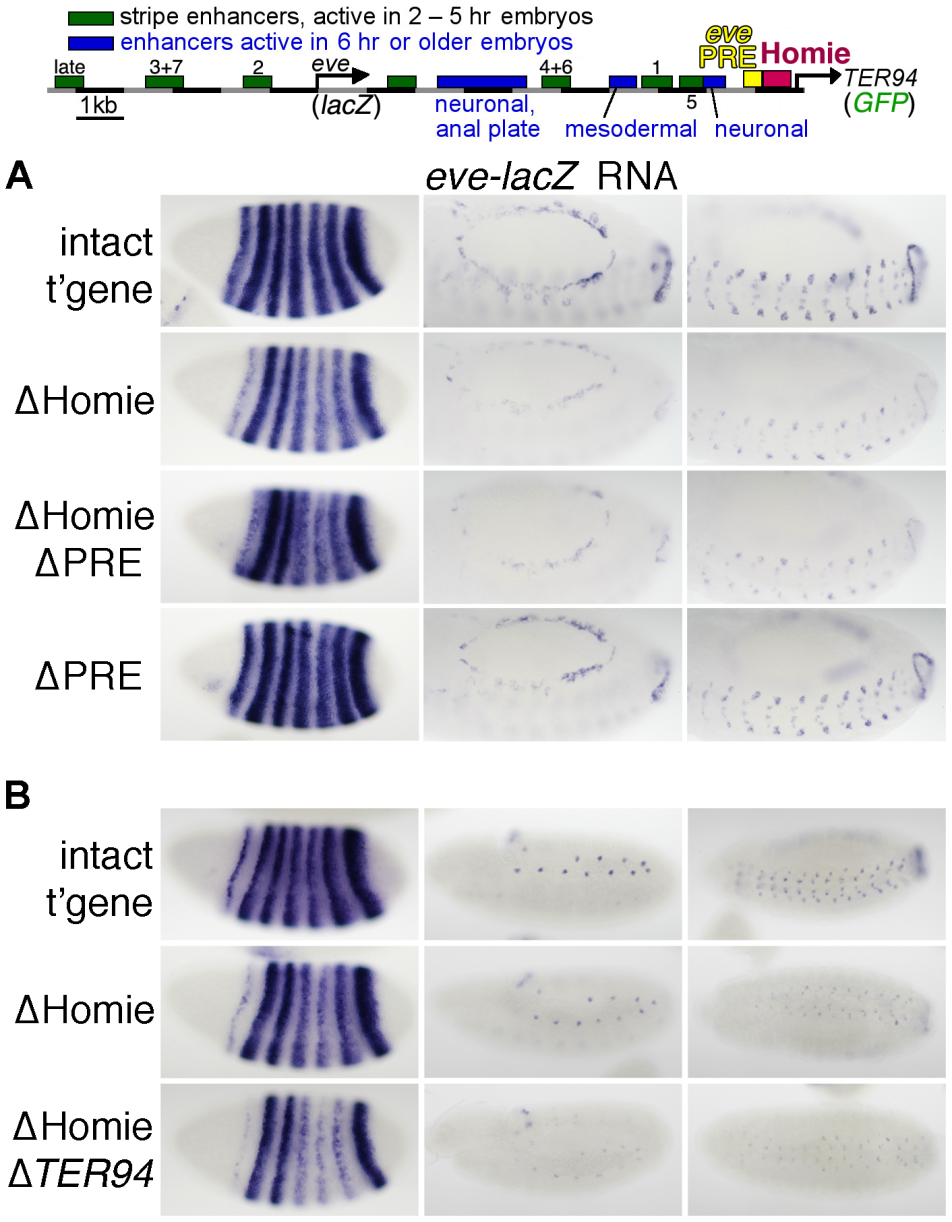
Homie facilitates *eve* 3′ enhancer action on the *eve* promoter through a mechanism that does not involve the *TER94* promoter. Expression of *lacZ* RNA driven by the *eve* promoter from the transgene diagrammed at the top (described in Figure 1A) and its derivatives was monitored by *in situ* hybridization. **A:** Representative embryos are shown at either stage 5 (left column) or stage 13 (middle and right columns, which show two different orientations and focal planes at higher magnification). Note that when Homie is deleted (“ΔHomie”), stripes 1, 4, 5, and 6 are weakened relative to stripes 2, 3, and 7 (left column), while all aspects of expression at later stages, in the mesoderm, CNS, and APR, are also weakened (middle and right columns). These weakened expression elements are all driven by enhancers located between the *eve* and *TER94* promoters. These effects are also seen when both Homie and the PRE are deleted (“ΔHomie ΔPRE”), but not when the PRE alone is deleted (“ΔPRE”). **B:** Representative embryos are shown at 3 embryonic stages (stages 5, 11, and 13, in the left, middle, and right columns, respectively). In ΔHomie Δ*TER94*, the entire *TER94-GFP* gene including the *TER94* promoter was removed. Note that the weakened activity of 3′ enhancers seen for ΔHomie is also seen for ΔHomie Δ*TER94*, indicating that competition with the *TER94* promoter is not causing the reduced *eve* promoter activity.

We then tested whether Homie affects *eve* promoter activity. To do this, we monitored transgenic *lacZ* expression, which is driven by the *eve* promoter (Figure 5, Figure S5A,B). When Homie is removed, there is a reduction in expression driven by enhancers located 3′ of the *eve* coding region. Interestingly, these are the *eve* enhancers located between Homie and the *eve* TSS. Comparing “ΔHomie” with the intact transgene at stage 5 (Figure 5 left column), we see that stripes 1, 4, 5, and 6 are weakened relative to stripes 2, 3, and 7. A similar reduction of expression is seen at later stages, where mesodermal, CNS, and APR expression are weakened by deletion of Homie (Figure 5 middle and right columns). This effect is seen at all transgene landing sites tested (Figure 5A, Figure S5A,B), although it varies in strength with the direction of transgene insertion (data not shown). Despite these differences, we consistently see significant disruptions of normal *eve* expression when Homie is removed.

It seemed possible that the effects of removing Homie on *eve* promoter activity were caused by the relief of enhancer blocking, which then might allow *eve* enhancers access to the *TER94* promoter. The resulting promoter competition might reduce *eve* expression. Alternatively, removing Homie might cause the loss of a chromosome conformation that favors *eve* enhancer-promoter interactions. This possibility is suggested by the ability of Homie to promote the activation by endogenous *eve* enhancers of a transgenic *eve* promoter located up to several megabases away [55]. To distinguish between these possibilities, we performed two sets of experiments. First, we tested whether expression from the *TER94* promoter occurs in a pattern that matches the loss of expression from the *eve* promoter when Homie is deleted. We found that this is not the case. Rather, *TER94* expression in an *eve* striped pattern does not show a difference among the stripes (Figure 1B ΔHomie, compare to Figure 5). Second, we directly tested the promoter competition hypothesis by deleting the *TER94* promoter in addition to Homie. Removing the potentially competing promoter did not restore normal *eve* expression (Figure 5B). While we cannot rule out competition with endogenous promoters, promoter competition seems unlikely to be the primary cause of the *eve* pattern disruptions that result from removal of Homie. The facilitation of *eve* promoter activity by Homie may therefore be due to its ability to organize specific chromosomal loops, possibly with the *eve* promoter (see Discussion).

Interestingly, heterologous insulators are able to restore normal *eve* enhancer-promoter interactions to different degrees (Figure S5C and data not shown), roughly in parallel to their abilities to restore PRE blocking (Figure 3A,B). For example, *gypsy* restores normal *eve* promoter activity, while *scs* does not (Figure S5C). The abilities of heterologous insulators to perform this function may be due to interactions between them and a region of the *eve* locus that normally interacts with Homie.

## Discussion

### A Transgenic *eve-TER94* Locus to Assess PRE and Insulator Activity

The *eve* 3′ insulator, Homie, was shown previously to have three activities: P-element transgene homing, enhancer blocking, and facilitation of long-range enhancer-promoter communication between endogenous *eve* enhancers and a transgenic promoter [55]. We sought to address how these activities relate to Homie's normal function. Both *eve* and *TER94* are essential genes, and *eve* is highly dose-dependent, making it problematic to manipulate the endogenous locus. Therefore, we constructed a transgene that contains these genes in their normal configuration. Both the *eve* and *TER94* coding regions were replaced with reporter genes to monitor promoter activity. This transgene simulates the expression pattern of both genes, when inserted at several different chromosomal sites. We used this system to manipulate both Homie and the nearby PRE, to assess their normal functions.

### Homie Protects the *TER94* Promoter from Spreading of PRE-dependent Repressive Chromatin

A major finding of this study is that Homie is required to prevent PRE-dependent repression of the *TER94* promoter. Removal of Homie causes a near-complete loss of the normally ubiquitous *TER94* promoter activity. Although Homie is close to the *TER94* promoter, its removal does not affect the promoter directly. Rather, removing Homie allows *eve* enhancers to drive the *TER94* promoter in an *eve* pattern (Figure 1). Furthermore, additional removal of the nearby PRE restores ubiquitous expression. This restoration is complete in some instances (e.g., Figures 1, 2), although it is incomplete in others (e.g., with a paternally-derived transgene in embryos, Figure S2). A simple explanation for the lack of complete restoration in some circumstances is that PRE activity varies in different tissues, and the *eve* 3′ PRE is partially redundant with other PREs at some times in development.

Ubiquitous expression of *TER94* in early embryos, as well as some of the later ubiquitous CNS expression, is due to maternally loaded RNA. Consistent with this, expression in ovaries is robust, and, like early embryonic expression, is strongly repressed without Homie (Figures 2, S4). Accompanying repression in both ovaries and embryos, trimethylation of H3K27 at *TER94-GFP* is strongly increased when Homie is removed (Figure 4). Thus, without Homie, the *eve* Pc domain spreads into the adjacent gene, apparently shutting down expression.

Homie is bound *in vivo* by most known insulator binding proteins, including Su(Hw), CP190, Mod(mdg4)67.2, BEAF32, CTCF, and GAF [27], [33]. In a previous study, depletion of CTCF by RNAi in a cultured cell line caused a reduction in H3K27me3 levels throughout the *eve* locus [36]. The authors suggested that depleting CTCF altered the activity of insulators flanking *eve*, which led to a decrease in H3K27me3. In contrast, we found that deletion of Homie did not cause a significant reduction in H3K27me3 levels in the *eve-lacZ* region of our pseudo-locus, either in embryos or in ovaries (Figure 4). There could be several possible reasons for this discrepancy, including the cell types assayed, and indirect effects of depleting CTCF.

### Tissue Specificity and Redundancy of PRE Activity

With removal of Homie, the spreading of H3K27me3 in ovaries is reversed by deletion of the PRE (Figure 4A,B). However, in embryos, this spreading is only partially reversed (Figure 4C,D). A simple explanation for this is that additional PRE activity within the *eve* locus comes into play in embryos. Consistent with this, the *eve* promoter-proximal region has PRE-like properties. It causes pairing-sensitive silencing of *mini-white* in transgenes that carry it [51], a property associated with most known PREs. Furthermore, it has consensus binding sites for several PRE-associated DNA binding proteins [8], [51], and it shares with the *eve* 3′ PRE the ability to support positive epigenetic maintenance of enhancer activity from embryos to larvae within *eve*-positive neurons [51].

Perhaps the clearest evidence for redundant PRE activity within the *eve* locus is that the level of H3K27me3 at the *eve-lacZ* coding region is not significantly reduced when the 3′ PRE is deleted. This is true in both embryos and ovaries. In contrast, spreading of the Pc domain into *TER94* in ovaries requires the 3′ PRE (Figure 4). Our data are consistent with the idea that PREs are the nucleation point for spreading of the H3K27me3 mark, and that PRE activity is regulated, so that PREs are differentially active in different tissues.

Furthermore, because there may be a dynamic balance between active and repressive chromatin, maintaining a boundary between them may have different requirements at different chromosomal locations, and at different times in development. Insulators that are not required to maintain a boundary in one cell type may be required for that function in other cells, or at specific times in development, as previously suggested [34]. One reason for such differences may be regulated PRE activity.

In some cases, spreading of repressive chromatin can be stopped by an active promoter [11], [64]. In the case of the *TER94* promoter, although it is robustly expressed, particularly in ovaries, this is not sufficient to stop the spreading of H3K27me3 in the absence of an insulator. This contrasts with the suggestion from recent genome-wide studies in both cultured cells and Drosophila that insulator protein function is generally not required to prevent spreading of H3K27me3 into active genes, or to maintain most normal gene expression [34], [36], [38]. Because many insulator proteins bind to overlapping sets of sites, it is likely that there is considerable redundancy in their function. Thus, knocking out any one of them may not reveal the full function of a majority of their binding sites.

### Enhancer Action from Within a Pc Domain

It is intriguing that the *eve* locus is a Pc domain with well-defined boundaries that flank its extensive regulatory regions. Within chromosomal domains of the Drosophila bithorax complex (BX-C), active enhancers prevent the establishment of repressive Pc-dependent chromatin in early embryos. Conversely, in tissues where such repressive chromatin has been established, such as in parts of the CNS and imaginal discs, later-acting enhancers are repressed [25]. Do similar mechanisms operate within the *eve* locus? Extensive dissection of *eve* regulatory DNA has not identified enhancers that can drive expression outside the normal *eve* pattern, arguing against such a close analogy with the BX-C. However, in PcG mutants, *eve* is ectopically expressed throughout the late-stage embryonic CNS [50], [54], showing that PcG genes do negatively regulate *eve*, as they do the Hox complexes.

In our previous studies of *eve* PRE activity, we found that in a transgenic context, both the 3′ PRE and the PRE-like *eve* promoter region could facilitate positive maintenance of an *eve* CNS enhancer from embryonic to larval stages, as well as prevent ectopic expression in cells that normally do not express *eve* [51]. Unlike maintenance elements [65] in the BX-C, the *eve* 3′ PRE was found to require the DNA binding PcG protein Pleiohomeotic rather than Trithorax-group members for positive maintenance [51]. In this study, we also see evidence of a positive effect of the *eve* 3′ PRE on enhancer activity. In this case, it facilitates *TER94-GFP* expression in an *eve* pattern when Homie is removed (Figure 1B: *eve*-like mesodermal and CNS expression are seen when Homie is removed, but are not seen when both Homie and the PRE are removed). One possible explanation for this is that *eve* enhancers have evolved to function within a Pc domain, and they may be better able to activate the *TER94* promoter when the Pc domain spreads over it. In this view, PREs facilitate both the on state and the off state, yet the chromatin may be differently modified in the two cases. This model is similar to the “integration model” proposed for how heterochromatin can have a positive effect on the expression of genes that normally reside within it [66].

### Homie's Activity Relative to that of Other Drosophila and Mammalian Insulators

Homie sits adjacent to the *eve* 3′ PRE, an arrangement that is reminiscent of some boundaries in the BX-C. The mammalian homologs of *eve*, *evx1* and *evx2*, are located at the 3′ end of the HOX-A and HOX-D clusters, respectively, suggesting that the ancestral *eve* locus was part of a Hox cluster [67]. Consistent with conservation of the *eve* insulator-PRE relationship, recent studies identified an enhancer-blocking activity between *evx2* and *Hoxd13* [68], and a PRE in the HOX-D cluster [69]. The presence of a PRE near an insulator, with a promoter on the other side, may indicate a functionally important boundary between active and repressive chromatin domains.

Previous studies showed that within the BX-C, neither *scs* nor *gypsy* could functionally replace *Fab-7* [70], indicating that there are different classes or strengths of insulators. In these cases, the primary effects of insulator deletion was ectopic activation, due to early acting enhancers (“initiators”) “turning off” PREs throughout a chromatin domain delineated by insulators [26], [71]. In our system, the major effect of insulator deletion is the spreading of the *eve* Pc domain, reminiscent of the shielding of transgenic reporter genes from repressive effects at some insertion sites [18], [72]–[75]. Despite the differences in normal function, BX-C insulators can replace Homie in our assay, indicating some degree of universality in insulator function as a PRE blocker. However, our assays did reveal differences in effectiveness in carrying out this function. Specifically, *scs'* showed slightly weaker activity than either *gypsy*, *Fab-7*, or *Fab-8*, while the activity of *scs* was highly orientation-dependent (Figure 3).

### Homie Blocks Enhancer-promoter Cross-talk, While Facilitating Enhancers that Lie Between It and the *eve* Promoter

Deletion of Homie results in expression of *TER94-GFP* in an *eve* pattern. In fact, the *eve* early embryonic stripe enhancers may access the *TER94* promoter even when Homie is present, because with a paternal-only transgene, we sometimes see *eve*-like stripe expression from *TER94-GFP* (Figure S2B “intact t'gene”). However, at later stages of embryogenesis, we do not see *eve*-like expression in either the mesoderm, CNS, or APR unless Homie is deleted. Therefore, one of Homie's functions is to prevent communication between the *TER94* promoter and *eve* enhancers.

Deletion of Homie, but not deletion of the PRE, also reduced *eve-lacZ* expression driven by the *eve* 3′ enhancers (Figures 5, S5). We considered the possibility that because the *TER94* promoter has access to *eve* enhancers in the absence of Homie, the resulting promoter competition might reduce *eve* promoter activity. However, in ΔHomie lines where we see *TER94* expressed in *eve* stripes, there is no apparent bias in expression toward the 3′ enhancers (Figures 1B, S1A), arguing against this possibility. Furthermore, at later embryonic stages, *eve* promoter activity is reduced when both Homie and the PRE are removed (in mesoderm, CNS, and APR, which are all the tissues where *eve* is expressed at these stages, Figure 5A), but this is not accompanied by *TER94-GFP* expression in an *eve* pattern (Figures 1B, S1A). Finally, when the *TER94* promoter is removed along with Homie, pattern disruptions persist (Figure 5B). While we cannot rule out competition with other promoters in the genome, these lines of evidence together suggest that promoter competition is unlikely to be responsible for this effect.

**Figure 6:**
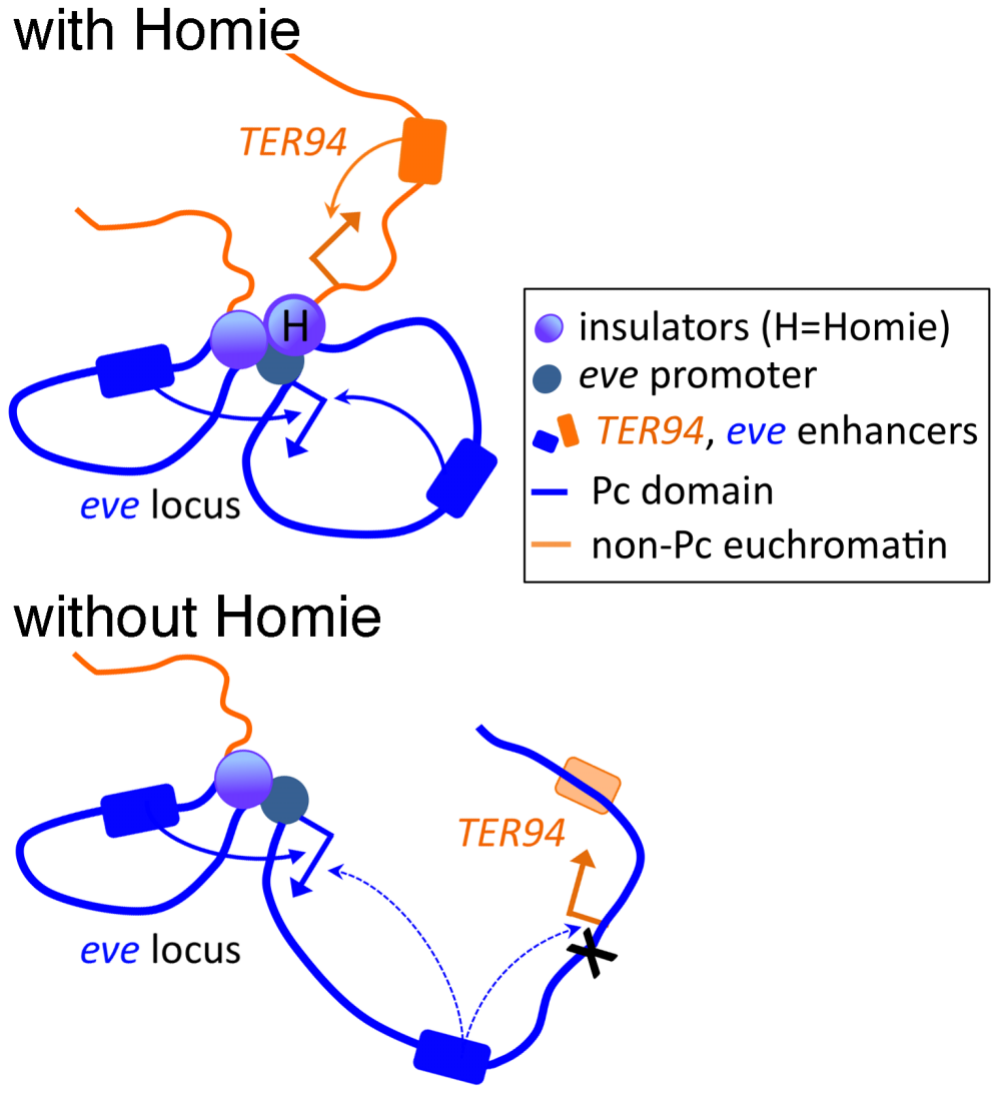
Model of the effects of Homie deletion on chromosome conformation and chromatin structure. At the top, Homie is present, and separates the *eve* Pc domain (blue) from the *TER94* locus, which is constitutively in active chromatin (orange). The *eve* enhancers both 5′ and 3′ of the *eve* start site efficiently activate the *eve* promoter, and the *TER94* enhancers activate the *TER94* promoter. Below, when Homie is removed, Pc-dependent chromatin spreads into the *TER94* locus, preventing its activation by *TER94* enhancers, but allowing *eve* enhancers to activate it. At the same time, *eve* 3′ enhancers interact with the *eve* promoter less efficiently, due to a change in chromosome conformation.

A second possible explanation for the reduction in *eve* 3′ enhancer-promoter communication when Homie is deleted is that a 3-dimensional (3-D) conformation that allows the *eve* promoter to better access the 3′ enhancers is stabilized by the presence of Homie. One possible conformation is a loop between the *eve* promoter region and Homie (Figure 6). Although we have not tested this directly, evidence consistent with this model is that activation of promoters, including the *eve* promoter, by downstream Gal4 binding sites can be facilitated by heterologous insulators in a model transgene assay [76]. This possible pairing of Homie with the *eve* promoter region would result in a loop that would bring the 3′ enhancers in closer proximity to the promoter. Such a model is similar to that proposed for the 3-D organization of regulatory regions upstream of the *Abd-B* gene [25]. If such loops are anchored to large clusters of insulator proteins, perhaps within insulator bodies, this may serve as a 3-D barrier that separates distinct chromatin domains, and occludes interactions between regulatory elements located on opposite sides of the insulator. At the same time, otherwise distant elements can be brought closer together, facilitating specific enhancer-promoter contacts, particularly if those elements are brought to the same side of the 3-D barrier.

### Implications for Other Insulators and PREs throughout the Genome

The activities of Homie and the *eve* PRE are largely interchangeable with those of other insulators and PREs, respectively, in our assay system. Previous studies showed that Homie and the *eve* PRE have the canonical properties of insulators and PREs when tested in other contexts [51], [55]. Thus, our results are likely to be applicable to many such elements throughout the genome. In particular, a common function of insulators is likely to be to limit the action of PRE-dependent repressive chromatin.

Genome-wide studies using RNAi to knock down specific insulator proteins suggested that insulators may not typically be required in their normal context either to block enhancer-promoter cross-talk or to prevent the spread of repressive chromatin [34], [36]. Our results suggest that Homie is critically important in its normal context for just such activities, functionally separating the loci on either side. Importantly, other insulators function in place of Homie. This suggests that the activities of insulators defined in model transgene assays do in fact correspond to their normal functions. In particular, as with Homie and the *TER94* promoter, the tendency of insulator proteins to cluster just upstream of promoters suggests that one of their typical functions is to shield basal promoters from the effects of upstream CRMs, especially PREs. Further, our finding that insulators facilitate enhancer-promoter communication in this context suggests that their ability to organize chromosomal conformations that augment appropriate transcription is also likely to be a common mode of endogenous insulator function.

## Materials and Methods

### Plasmid Construction and Transgene Production

The *eve-TER94* locus construct (“intact t'gene” in figures) was created as follows (detailed sequence coordinates are given in Figure S6). DNA from −6.4 kb to +166 bp relative to the *eve* TSS was fused to the *lacZ* coding region. The 3′ end of the *lacZ* coding region was fused to DNA from +1.3 to +11.4 kb, which includes the *eve* poly-A signal, and extends into the 3rd exon of *TER94*. This was joined with the *EGFP* coding region, followed by the poly-A signal of α–*tubulin*. The entire construct was placed between two inverted attB sequences [63], [77]. The following deletions were then made in this construct: from +8.4 to +9.2 kb for ΔPRE, from +8.4 to +9.7 kb for ΔHomie ΔPRE, and from +9.2 to +9.7 kb for ΔHomie. To test promoter competition between *eve* and *TER94*, DNA from −7.4 to +8.6 kb relative to the *eve* TSS was used, with the *eve* coding region replaced by that of *lacZ*, as described above. This construct does not contain the *TER94* promoter.

Replacements of Homie with either heterologous insulators or phage λ DNA were created using the ΔHomie construct, and adding DNA fragments corresponding to *gypsy* [78], *Fab-7* [79]–[82], *Fab-8* [83], [84], *scs* [85], [86], *scs'* [85], [86], or λ DNA (see Figure S6 for details). For testing repression activity of heterologous PREs, either the *engrailed* 181PRE [87] or the *bxd* PRE [88] were inserted into the ΔHomie ΔPRE construct at the site of deletion. For testing Homie activity against these PREs, either the *en* PRE or the *bxd* PRE were inserted into the ΔPRE construct at the site of deletion.

All transgenic lines were made using φC31 recombinase-mediated cassette exchange (RMCE) [63]. Three alternative attP target sites were used, at cytological locations 95E5, 74A2, and 30B5. The direction of each insertion was determined by PCR. Both directions were analyzed if obtained. Some variations with insertion site were found, as described in Results.

### Analysis of Gene Expression in Embryos and Ovaries

Embryos were collected at time points described in figure legends, and subjected to *in situ* hybridization using DIG-labeled anti-sense RNA probes against either *lacZ* or *GFP*. Expression patterns were visualized by alkaline phosphatase-conjugated anti-DIG with BCIP and NBT as substrates (Roche Applied Science).

GFP expression was detected by fluorescence microscopy in ovaries dissected from 1–2 day-old females. In some cases, expression was also detected using anti-GFP antibody staining (Roche Applied Science), analyzed by confocal microscopy (Zeiss) of material in DAPI-containing mounting medium.

### Chromatin Immunoprecipitation

Ovaries were dissected from 2–3 day-old females. Fifty ovaries were cross-linked in 1.8% formaldehyde in PBS for 10 min. After sonication so as to produce a peak near 500 bp in the DNA fragment size distribution, isolated chromatin was immunoprecipitated with anti-H3K27me3 (EMD Millipore), and with rabbit IgG (Jackson ImmunoResearch) as a negative control. Precipitated chromatin samples were collected using ProteinG magnetic beads (EMD Millipore). Immunoprecipitated DNA samples were dissolved in 20–50 µl TE, and 1 µl was used for each PCR reaction. Either duplicate or triplicate samples were analyzed by real-time PCR (Life Technologies, StepOnePlus), using SYBR Green Master Mix with ROX dye (Roche Applied Science). Data were analyzed with StepOne software (Life Technologies), using the standard curve method. Standard deviations were calculated using Excel software (Microsoft). Embryo ChIP analysis was described previously [51], except that results were quantified by real-time PCR, as described above for ovary analysis.

Specific ChIP signals were determined by subtracting the average non-specific IgG signal from the average α-H3K27me3 signal, with standard deviations combined by adding. Errors bars for specific signals relative to that of endogenous *eve* were determined by adding the relative errors in quadrature; that is, by taking the sum of the squares of the relative standard deviations (the standard deviations divided by their respective averages) to give the square of the relative standard deviation of the ratio.

The following primers were used: TCCAGTCCGGATAACTCCTTGAAC and TGTAGAACTCCTTCTCCAAGCGAC for the endogenous *eve* coding region, TGAAGCCACCGCGTGGTATTCTTA and TTTGGACATGATCTCCGGTCCGTT for the endogenous *TER94* coding region, GCTGTGCCGAAATGGTCCATCAAA and TACTGACGAAACGCCTGCCAGTAT for the transgenic *eve-lacZ* coding region, and GGGCACAAGCTGGAGTACAACTACAA and TGGCGGATCTTGAAGTTCACCTTG for the transgenic *TER94-GFP* coding region.

### RT-PCR

Total RNA was purified from either five pairs of ovaries from 2–3 day-old females or 10–20 µl of dechorionated embryos for each data point, using an RNA purification kit (Roche Applied Science). RNA was eluted in 50–100 µl elution buffer and stored at −80°C. cDNA was synthesized using the Transcriptor first strand cDNA synthesis kit (Roche Applied Science), and quantified by real-time PCR as described above. A constitutively expressed RNA, *RpL32* (a.k.a. *RP49*), was used to normalize GFP RNA levels. The primers listed above for *TER94-GFP* were used for GFP, and AAGCCCAAGGGTATCGACAACAGA and TGCACCAGGAACTTCTTGAATCCG were used for *RpL32*.

## Supporting Information

Figure S1 Homie shields the *TER94* promoter from *eve* PRE activity. **A, B:** similar effects on *TER94* promoter activity are seen at two chromosomal landing sites (different from that used in Figure 1), where deletion of Homie causes a loss of ubiquitous activity, and further deletion of the PRE restores ubiquitous expression. Some differences are seen at the landing site shown in B, mostly attributable to a lower expression level from all three transgenes, which makes the *eve*-like expression (visible in the other lines when Homie is deleted) difficult to detect. The attP sites used are at cytological locations 74A2 (A) and 30B5 (B). A longer staining reaction time than that used in B, “ΔHomie”, showed both expression in stripes and in *eve*-expressing cells of the mesoderm (not shown).
(TIF)

Figure S2 When the transgenic *eve-TER94* locus is heterozygous and paternally derived, embryonic *TER94-GFP* expression is repressed in the absence of Homie as it is in ovaries, but this repression is not dependent on the 3′ PRE. Expression of GFP RNA in the transgenic lines indicated on the left from two different transgene landing sites (in A and B) is visualized at 3 different embryonic stages (indicated along the top) by *in situ* hybridization. The levels of GFP RNA (extracted from 2–3 hr-old embryos), quantified in triplicate, are show on the right (averages with standard deviations, see Materials and Methods). **A:** In the top 3 rows and in the graph, expression is exclusively zygotic (mothers did not carry a transgene; the paternal transgene is at the same insertion site, 95E5, as that shown in Figure 1), while the bottom row shows, for comparison, maternal plus zygotic expression (both mothers and fathers were homozygous for the transgene) when Homie is deleted. Note that without Homie, the expression pattern is similar (albeit weaker) in heterozygotes as in homozygotes, consistent with the maternal contribution being repressed in the absence of Homie. This similarity includes *eve*-like stripes at all 3 stages, as well as *eve*-like mesodermal expression (dots in the dorsal-most part of each segment near the center of the germ-band extended embryo at stage 11). The quantitation shown in the graph suggests that ubiquitous zygotic expression is repressed when Homie is deleted. However, unlike for maternally derived expression (as shown in Figures 1, S1, and 2), this repression is not dependent on the PRE. This is consistent with there being other PREs that are active in embryos, possibly within the transgenic *eve* locus (see Results and Discussion). **B:** zygotic expression from a second transgene landing site (cytological location 74A2). Note that at this landing site, *eve*-like stripe expression is present from the intact transgene, indicating that some *eve* enhancers can work on the *TER94* promoter when Homie is present, at least at some landing sites. Quantitation, shown in the graph, indicates that overall zygotic expression is reduced when Homie is deleted, consistent with repression of ubiquitous expression driven by *TER94* enhancers present in the transgene. As with the other landing site, this repression is not dependent on the PRE, but may be due to redundant PRE activity.
(TIF)

Figure S3 *TER94-GFP* is expressed throughout oogenesis in both germ cells and somatic epithelial follicle cells. Ovarioles dissected from female flies (carrying transgenic *TER94-GFP* in the context of the transgene diagrammed in Figure 1A inserted at cytological location 95E5) aged 24–48 hours after eclosion were stained using antibodies to GFP (red) and DAPI (blue). Individual egg chambers were optically sectioned using confocal microscopy. Imaging of the surface (left column) shows mostly ovarian follicle cells, while imaging of the interior (right column) shows ovarian follicle cells at the periphery, and nurse cells and the oocyte in the central region. Note that GFP is strongly expressed in all cells from the intact transgene (top row), but not significantly above background in any cells when Homie is deleted from the transgene (bottom row).
(TIF)

Figure S4 Homie blocks PRE action in ovaries. Fluorescence in ovarioles from transgenic lines carrying the indicated transgenes, at two different chromosomal insertion sites (left column, cytological location 74A2; right column, 30B5), distinct from that shown in Figure 2 (corresponding to those shown in Figure S1). Note the same trend, wherein deletion of Homie causes severe repression of fluorescence from transgenic *TER94-GFP*, while additional deletion of the PRE causes restoration of *TER94* promoter activity. The graph at the bottom shows, on a log scale, the results of quantitation, as in Figure 2, of GFP RNA from ovaries of the line shown above it in the same column. Note that GFP RNA levels decrease about 1000-fold when Homie is deleted, and are partially restored (about 200-fold) by additional deletion of the PRE.
(TIF)

Figure S5 Homie facilitates *eve* 3′ enhancer action on the *eve* promoter. Expression of *lacZ* RNA driven by the *eve* promoter from the transgene diagrammed in Figure 1A and its derivatives was monitored by *in situ* hybridization. **A, B:** results from two different transgene landing sites (cytological locations 74E2 and 30B5, respectively), distinct from that shown in Figure 5A. Representative embryos at three stages are shown. Note that when Homie is deleted (“ΔHomie”), stripes 1, 4, 5, and 6 are weakened relative to stripes 2, 3, and 7 (left column, stage 5). This effect is still seen when both Homie and the PRE are deleted (“ΔHomie ΔPRE”), and occurs at both landing sites. In addition, mesodermal expression at stage 11 (middle column), as well as CNS and APR expression at stage 13 (right column), are weakened when Homie is deleted. These effects also persist when both Homie and the PRE are deleted, and are seen at both landing sites. **C:** results from two of the Homie-replacement lines shown in Figure 3. Note that *gypsy*, but not *scs*, facilitates 3′ enhancer activity.
(TIF)

Figure S6 Construct details. In **A** are listed the details of construction of the *eve-TER94* pseudo-locus transgene and it primary derivatives, as described in Materials and Methods. In **B** are listed the details of modifications to that construct, as described in Materials and Methods, Results, and figure legends.
(TIF)
