# Supplementary figures and images for: The Drosophila eve Insulator Homie Promotes eve Expression and Protects the Adjacent Gene from Repression by Polycomb Spreading
Source: PLoS Genet. 2013 Oct 31;9(10):e1003883. doi: 10.1371/journal.pgen.1003883 (PMC3814318; doi:10.1371/journal.pgen.1003883)

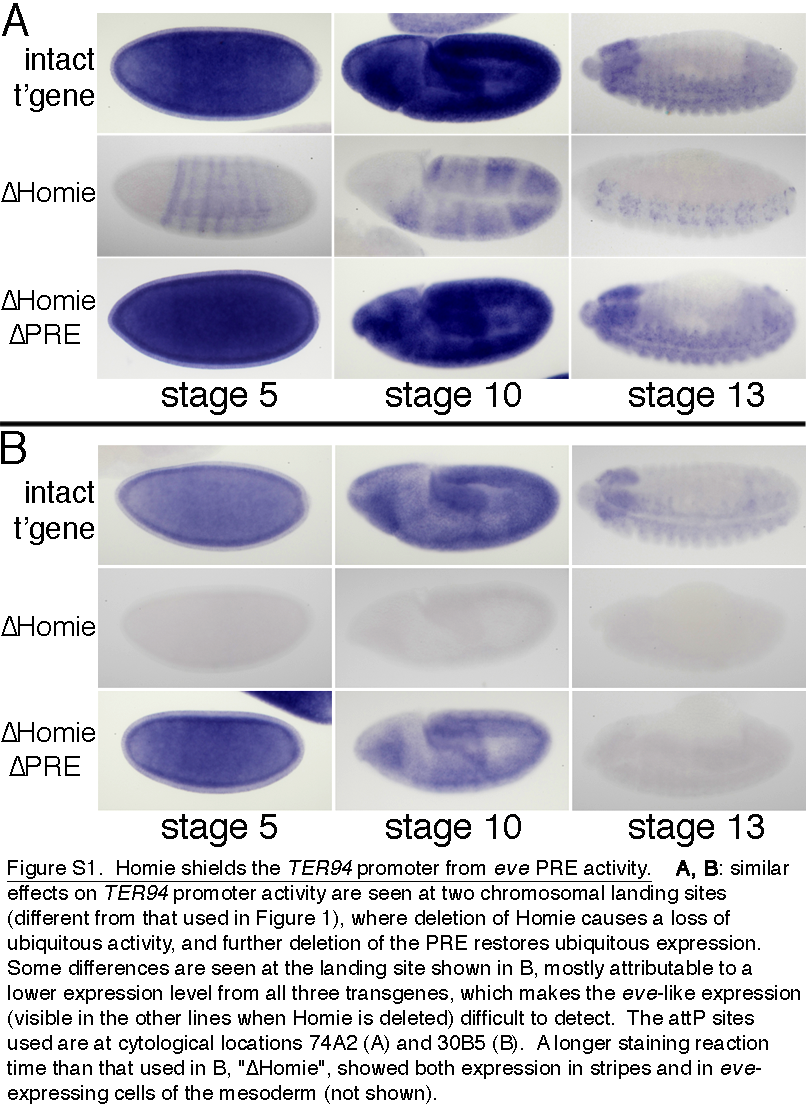

Supplement: Figure S1 — Homie shields the TER94 promoter from eve PRE activity. A, B: similar effects on TER94 promoter activity are seen at two chromosomal landing sites (different from that used in Figure 1), where deletion of Homie causes a loss of ubiquitous activity, and further deletion of the PRE restores ubiquitous expression. Some differences are seen at the landing site shown in B, mostly attributable to a lower expression level from all three transgenes, which makes the eve-like expression (visible in the other lines when Homie is deleted) difficult to detect. The attP sites used are at cytological locations 74A2 (A) and 30B5 (B). A longer staining reaction time than that used in B, “ΔHomie”, showed both expression in stripes and in eve-expressing cells of the mesoderm (not shown). (TIF) [file pgen.1003883.s001.tif]

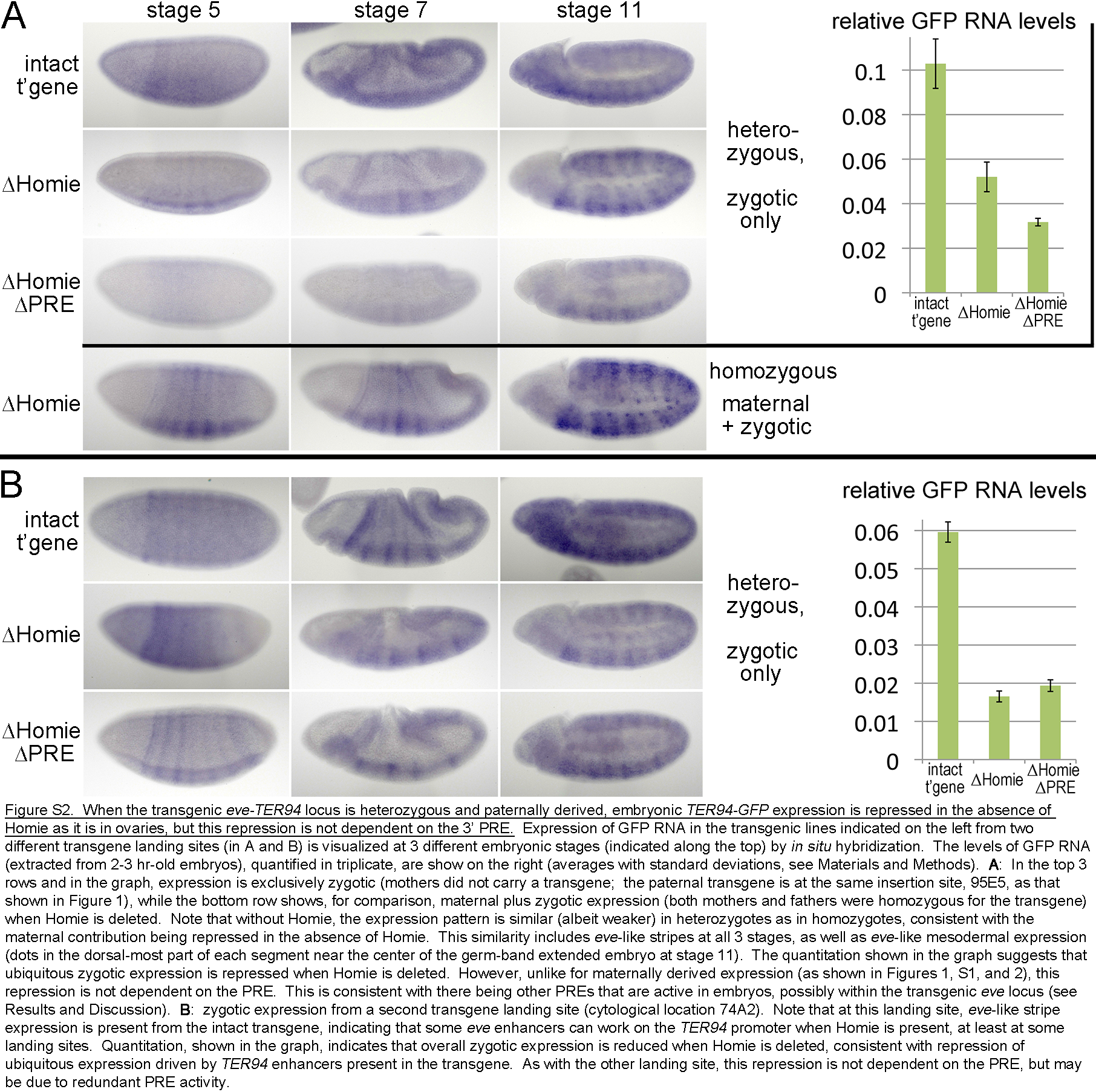

Supplement: Figure S2 — When the transgenic eve-TER94 locus is heterozygous and paternally derived, embryonic TER94-GFP expression is repressed in the absence of Homie as it is in ovaries, but this repression is not dependent on the 3′ PRE. Expression of GFP RNA in the transgenic lines indicated on the left from two different transgene landing sites (in A and B) is visualized at 3 different embryonic stages (indicated along the top) by in situ hybridization. The levels of GFP RNA (extracted from 2–3 hr-old embryos), quantified in triplicate, are show on the right (averages with standard deviations, see Materials and Methods). A: In the top 3 rows and in the graph, expression is exclusively zygotic (mothers did not carry a transgene; the paternal transgene is at the same insertion site, 95E5, as that shown in Figure 1), while the bottom row shows, for comparison, maternal plus zygotic expression (both mothers and fathers were homozygous for the transgene) when Homie is deleted. Note that without Homie, the expression pattern is similar (albeit weaker) in heterozygotes as in homozygotes, consistent with the maternal contribution being repressed in the absence of Homie. This similarity includes eve-like stripes at all 3 stages, as well as eve-like mesodermal expression (dots in the dorsal-most part of each segment near the center of the germ-band extended embryo at stage 11). The quantitation shown in the graph suggests that ubiquitous zygotic expression is repressed when Homie is deleted. However, unlike for maternally derived expression (as shown in Figures 1, S1, and 2), this repression is not dependent on the PRE. This is consistent with there being other PREs that are active in embryos, possibly within the transgenic eve locus (see Results and Discussion). B: zygotic expression from a second transgene landing site (cytological location 74A2). Note that at this landing site, eve-like stripe expression is present from the intact transgene, indicating that some eve enhancers can work on the [file pgen.1003883.s002.tif]

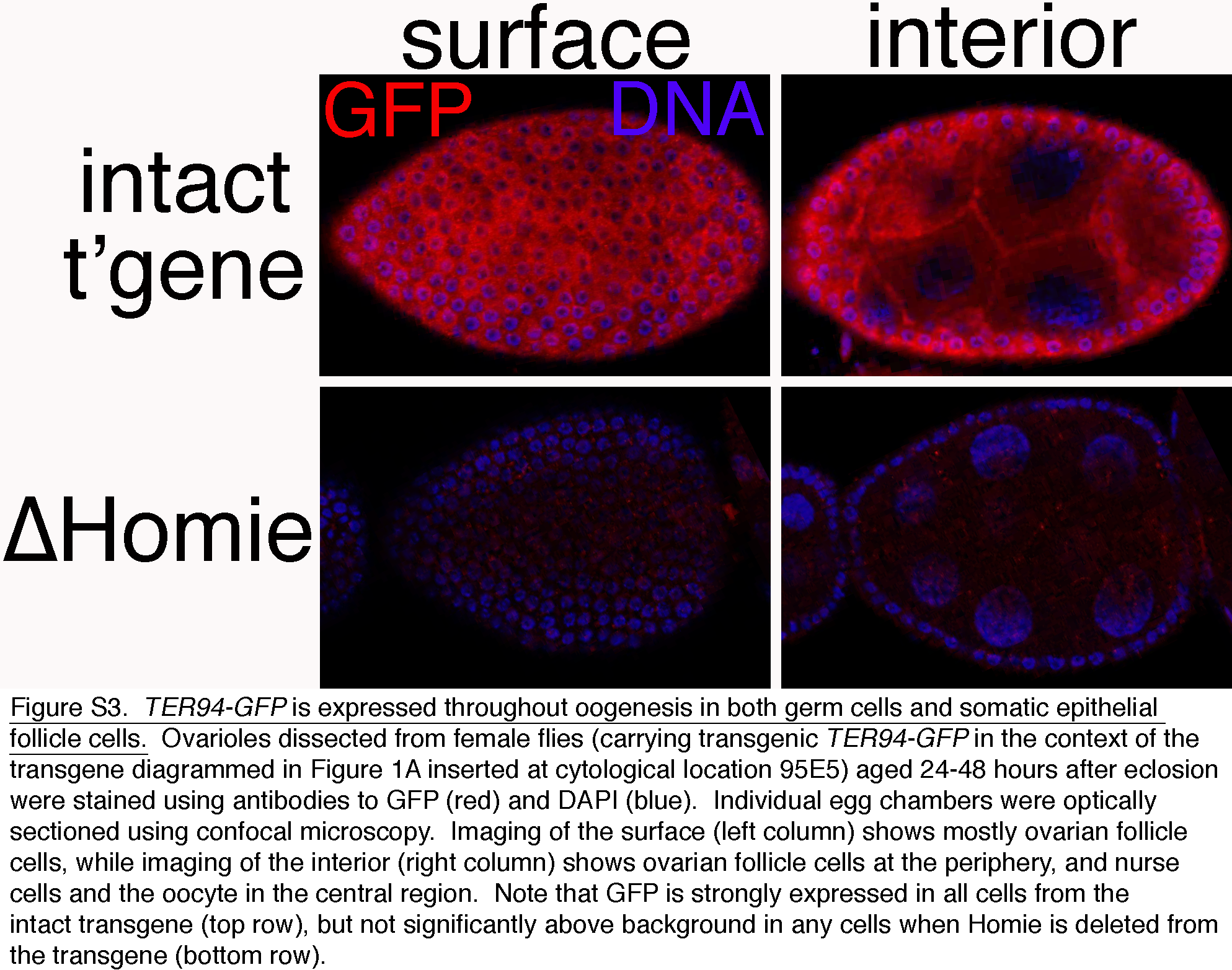

Supplement: Figure S3 — TER94-GFP is expressed throughout oogenesis in both germ cells and somatic epithelial follicle cells. Ovarioles dissected from female flies (carrying transgenic TER94-GFP in the context of the transgene diagrammed in Figure 1A inserted at cytological location 95E5) aged 24–48 hours after eclosion were stained using antibodies to GFP (red) and DAPI (blue). Individual egg chambers were optically sectioned using confocal microscopy. Imaging of the surface (left column) shows mostly ovarian follicle cells, while imaging of the interior (right column) shows ovarian follicle cells at the periphery, and nurse cells and the oocyte in the central region. Note that GFP is strongly expressed in all cells from the intact transgene (top row), but not significantly above background in any cells when Homie is deleted from the transgene (bottom row). (TIF) [file pgen.1003883.s003.tif]

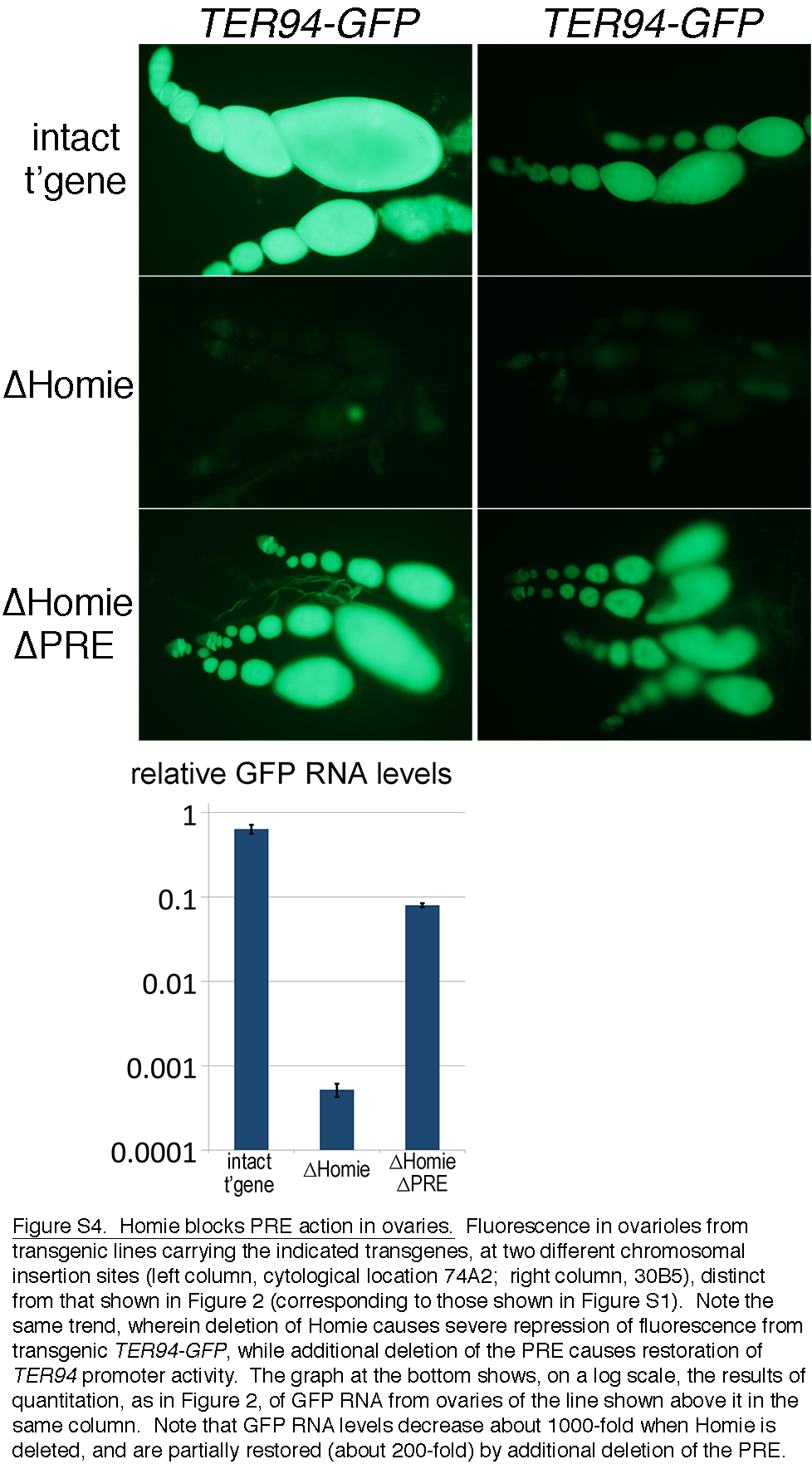

Supplement: Figure S4 — Homie blocks PRE action in ovaries. Fluorescence in ovarioles from transgenic lines carrying the indicated transgenes, at two different chromosomal insertion sites (left column, cytological location 74A2; right column, 30B5), distinct from that shown in Figure 2 (corresponding to those shown in Figure S1). Note the same trend, wherein deletion of Homie causes severe repression of fluorescence from transgenic TER94-GFP, while additional deletion of the PRE causes restoration of TER94 promoter activity. The graph at the bottom shows, on a log scale, the results of quantitation, as in Figure 2, of GFP RNA from ovaries of the line shown above it in the same column. Note that GFP RNA levels decrease about 1000-fold when Homie is deleted, and are partially restored (about 200-fold) by additional deletion of the PRE. (TIF) [file pgen.1003883.s004.tif]

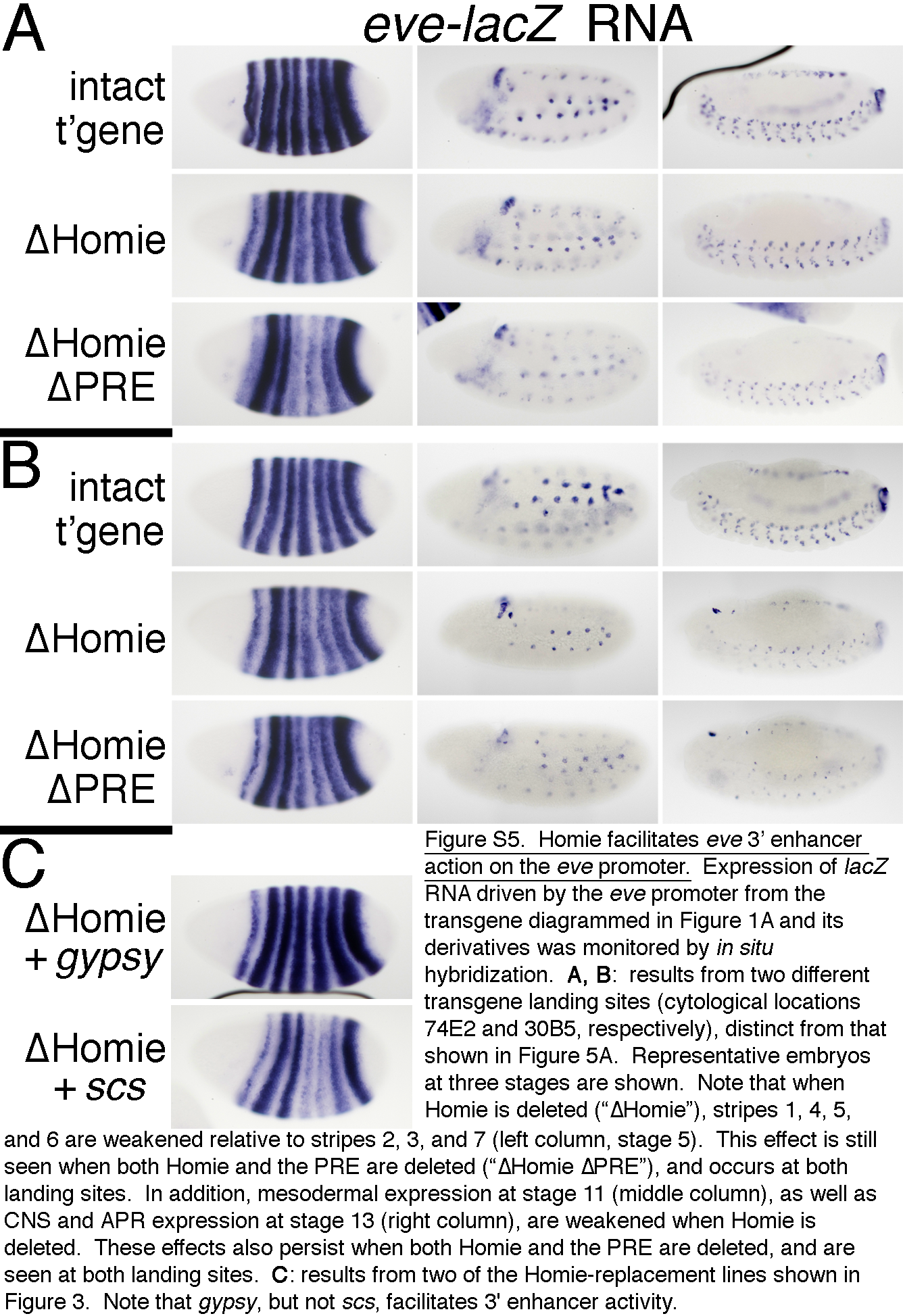

Supplement: Figure S5 — Homie facilitates eve 3′ enhancer action on the eve promoter. Expression of lacZ RNA driven by the eve promoter from the transgene diagrammed in Figure 1A and its derivatives was monitored by in situ hybridization. A, B: results from two different transgene landing sites (cytological locations 74E2 and 30B5, respectively), distinct from that shown in Figure 5A. Representative embryos at three stages are shown. Note that when Homie is deleted (“ΔHomie”), stripes 1, 4, 5, and 6 are weakened relative to stripes 2, 3, and 7 (left column, stage 5). This effect is still seen when both Homie and the PRE are deleted (“ΔHomie ΔPRE”), and occurs at both landing sites. In addition, mesodermal expression at stage 11 (middle column), as well as CNS and APR expression at stage 13 (right column), are weakened when Homie is deleted. These effects also persist when both Homie and the PRE are deleted, and are seen at both landing sites. C: results from two of the Homie-replacement lines shown in Figure 3. Note that gypsy, but not scs, facilitates 3′ enhancer activity. (TIF) [file pgen.1003883.s005.tif]

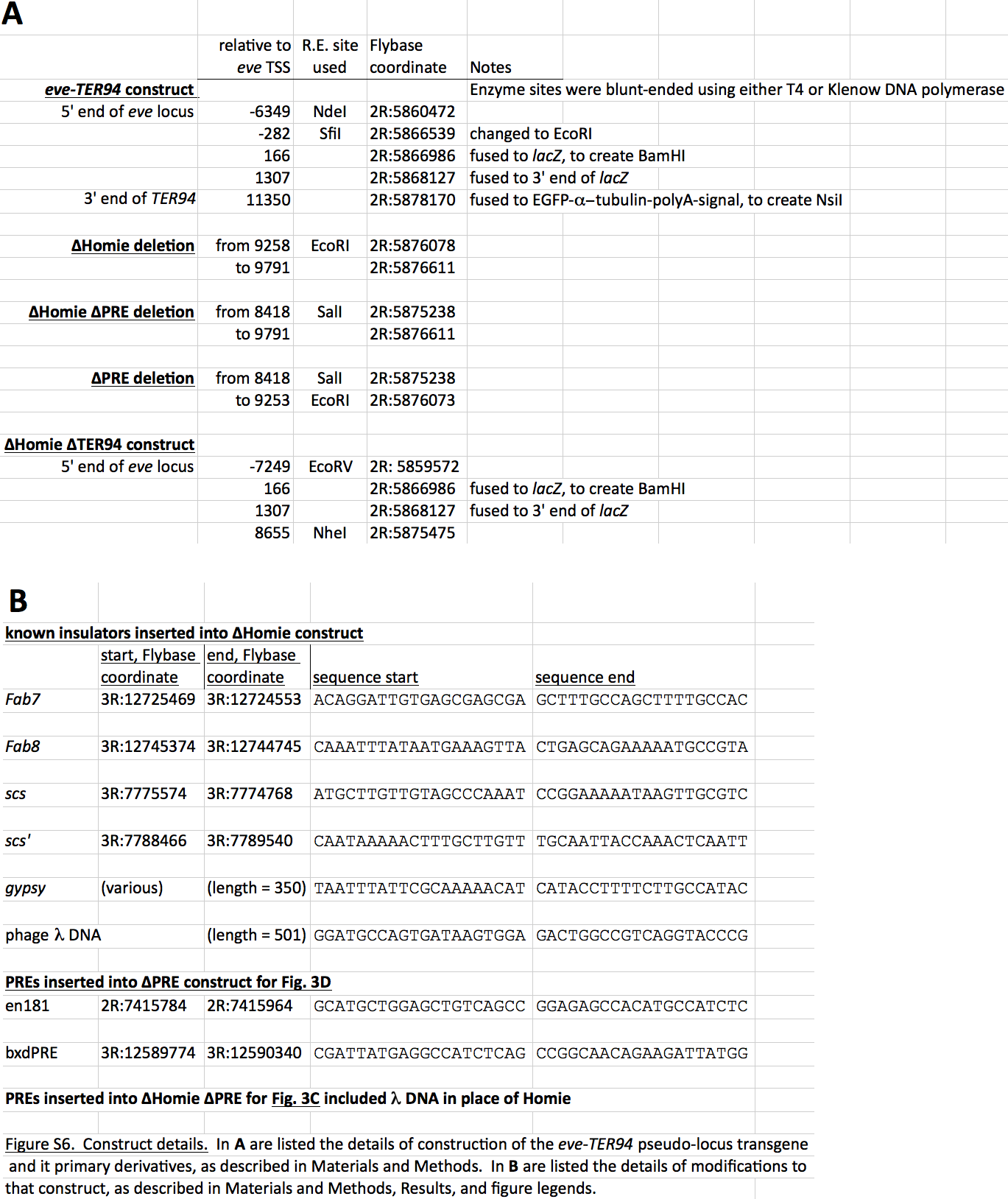

Supplement: Figure S6 — Construct details. In A are listed the details of construction of the eve-TER94 pseudo-locus transgene and it primary derivatives, as described in Materials and Methods. In B are listed the details of modifications to that construct, as described in Materials and Methods, Results, and figure legends. (TIF) [file pgen.1003883.s006.tif]
